# Death of a Pulp from Abscess of an Adjoining Tooth

**Published:** 1870-08

**Authors:** W. E. Driscoll


### DEATH OF A PULP FROM ABSCESS OF ADJOINING TOOTH.

BY W. E. DRISCOLL.

I have often thought that an alveolar abscess arising from the root of one tooth endangers the pulp of the adjoining ones, of either side, but have never had so good an illustration of the *fact* before, as is afforded by the two teeth I send with this; left superior central and lateral incisors. A plug was lost from the central, near three years ago, and an abscess was established about two years ago. Had nothing done for it until it was extracted, to-day. A second abscess had an opening at a point between the apices of the lateral incisor and the cuspid. There was no decay of either of the last two mentioned teeth. But the central incisor was loose, and very sore. I concluded that the abscess of the central had caused the death of the branch of the nerve leading from the lateral to the main branch, although the ulceration did not extend quite so far. This dead branch needing some outlet for the pus it produced, found its easiest egress at the point before stated. The patient not consenting to treatment necessary to filling, they were extracted, and found to be as first supposed. The apex of the lateral incisor was wasted away by its abscess, about one-eighth of an inch.

---

Editor Dental Register:—I wish to call attention to a peculiar case of neuralgia, of the superior maxillary, which came under my treatment. Nearly two years ago a lady called at my office and consulted me in reference to a neuralgic affection of the right side of the superior maxillary. The pain was greatest at the side of the two bicuspids and first molar, or in that region. All the teeth had been extracted nearly four years previously; during two years of that time her sufferings were extremely severe. Thinking, however, that there might be a remaining root, which escaped observation, I made an incision the whole length of the space occupied by the two bicuspids and first molar, and dissected the soft parts down, and with a suitable chisel and a pair of bone forceps, cut away a considerable amount of the process down to the base of the jaw. Not finding what I was in search of I dressed the wound, and to my surprise I found the lady entirely relieved from all neuralgic pain of the face and head. I have seen her twice since the operation, and she informs me that she has never had a recurrence of the disease. And as I think it might be of some importance to the Dental profession I report the case; and would refer to an article in the July number, 1870, of the *American Journal of Medical Science*; by S. D. Gross, M. D., Professor of Surgery in the Jefferson Medical College, of Philadelphia, who has reported several cases of neuralgia of the jaw, which he has treated by surgical operations, which has been crowned with perfect success.

However, as I have another case of nearly the same kind, which I intend treating by a surgical operation, I will close this article, and report the case in due time.

C. C. BURNS.

